# DHAT SYNDROME: A REAPPRAISAL

**DOI:** 10.4103/0019-5154.49002

**Published:** 2009

**Authors:** Vandana Mehta, Abhishek De, C Balachandran

**Affiliations:** *From the Department of Skin and STD, Kasturba Medical College, Manipal, Karnataka, India. E-mail: vandanamht@yahoo.com*

Sir,

Dhat syndrome is a true culture bound sex neurosis quite common in natives of the Indian subcontinent. Culture bound syndromes (CBS) were defined by Littlewood and Lipsedge as ‘episodic and dramatic reactions specific to a particular community.’[1] In 1969, Yap coined the term CBS to delineate a rare and exotic group of disorders that cause little damage to humanity; however, they may consist of unpredictable and chaotic behavior.[2]

The term ‘Dhat’ gets its origin from the Sanskrit word ‘Dhatus’, which, according to the Susruta Samhita, means elixir that constitutes the body. It was first described in western psychiatric literature by Wig, with vague psychosomatic symptoms of fatigue, weakness, anxiety, loss of appetite, guilt and sexual dysfunction, attributed by the patient to loss of semen in nocturnal emission, through urine or masturbation.[3]

Ayurvedic literature describing semen as a vital constituent of the human body dates back to 1500 BC. The disorders of ‘Dhatus’ have been elucidated in the Charak Samhita, which describes a disorder called ‘Shukrameha’ in which there is a passage of semen in the urine. Similar conditions have been described under various names from China (Shen K'uei), Sri Lanka (Prameha) and other parts of South East Asia (Jiryan). Malhotra and Wig called ‘Dhat’ ‘a sexual neurosis of the Orient’.[4] In China, anxiety following semen loss (Shen-K'uie) has been associated with epidemics of Koro, which is another culture bound syndrome in which the individual holds the belief that his penis is shrinking into his body and disappearing. Tissot's paper in 18th century stating that even an adequate diet could waste away through seminal emission gained popularity amongst the emerging middle class and led Western Europe to an era of masturbating insanity.

The International Classification of diseases ICD-10 classifies Dhat syndrome as both a neurotic disorder (code F48.8) and a culture specific disorder (Annexe 2) caused by ‘undue concern about the debilitating effects of the passage of semen.’ It is a commonly recognized clinical entity in India and South East Asia and is also widespread in Nepal, Sri Lanka, Bangladesh and Pakistan.

Dhat Syndrome is characterized primarily with complaints of loss of semen through urine, nocturnal emission or masturbation, accompanied by vague symptoms of weakness, fatigue, palpitation and sleeplessness. The condition has no organic etiology. It may sometimes be associated with sexual dysfunction (impotence and premature ejaculation) and psychiatric illness (depression, anxiety neurosis or phobia).[5]

Dhikav *et al*.[6] studied 30 patients with Dhat syndrome and found that the mean age of onset was 19 years, with mean duration of the illness being 11 months. Twenty out of 30 patients met the diagnostic criteria for depression. A majority of the cases were unmarried (64.2%) and educated till 5th standard or above. Ten patients (33.33%) were found to have a co-morbid problem of premature ejaculation and ten patients (6.6%) reported erectile dysfunction. Bhatia and Malik[7] studied 93 patients with Dhat syndrome and found weakness (70.8%) to be the most common complaint, followed by fatigue, palpitation, sleeplessness, loss of interest, loss of concentration, depression and headache. Among the psychiatric problems, neurotic depression was found to be the most common, found in 39.5%, followed by anxiety neurosis in 20.8%, major depressive psychosis in 6.3% and phobia in 2.1%. In 18.6% of the patients, there was associated suicidal tendency.

Randomized clinical trials suggest that the most effective clinical management of this condition lies in a combination of anti-anxiety and antidepressant medications, with counseling and cognitive behavioral therapy. In the past few months, we had also encountered many such patients in our Out Patient Department, who mainly presented with anxiety and fear of loosing a vital component of their body. Most of them required psychiatric referral and benefited in the long run from anti-anxiety drugs, with behavioral therapy. In 1998, Bhatia *et al*. compared the treatment response in four different groups of patients and found the maximum improvement in the group treated with anti-anxiety medications (58%).[8]

In conclusion, Dhat syndrome is a very common culture bound sex neurosis, widely prevalent in India. Though the origin of this condition is deeply rooted to the overvalued role of semen as a vital substance of the human body, sexual awareness and improved literacy rates have still not been able to convince the general population of its non organic nature. Though primarily a psychiatric diagnosis, dermatology postgraduates should be aware of this clinical entity as a part of their curriculum.

## References

[1] Lipsedge M, Littlewood R, Granville-Grosman (1985). Transcultural psychiatry. Recent advances in clinical psychiatry.

[2] Yap PM, Caudil W, Lin T (1969). The culture bound reactive syndrome. Mental health research in Asia and the pacific.

[3] Wig N (1960). Problems of mental health in India. J Clin Soc Psychiatry.

[4] Malhotra HK, Wig NN (1975). Dhat syndrome: A culture bound sex neurosis of the orient. Arch Sex Behav.

[5] Sumathipala A, Siribaddana SH, Bhugra D (2004). Culture bound syndromes: The story of Dhat syndrome. Br J Psychiatry.

[6] Dhikav V, Aggarwal N, Gupta S, Jadhavi R, Singh K (2008). Depression in Dhat syndrome. J Sex Med.

[7] Bhatia MS, Malik SC (1991). Dhat syndrome: A useful diagnostic entity in Indian culture. Br J Psychiatry.

[8] Bhatia MS, Choudhary S (1998). Dhat syndrome: A culture bound sexual neurosis. Indian J Med Sci.

